# Unveiling an Unusual Cause of Cardiac Tamponade

**DOI:** 10.7759/cureus.50984

**Published:** 2023-12-23

**Authors:** Inês Conde, Nuno Salomé, Alexandra Sousa, Catarina Quina

**Affiliations:** 1 Cardiology, Hospital de Braga, Braga, PRT; 2 Cardiology, Hospital of Santa Maria Maior, Barcelos, PRT

**Keywords:** cardiac tamponade, atrial septal defects occluder, transcatheter occluder devices, atrial septal defect secundum, pericardial effusion. cardiac tamponade

## Abstract

This case presents a 48-year-old woman initially diagnosed with an ostium secundum atrial septal defect (ASD) at the age of 36. Twelve years post-intervention, she presented to the emergency department with cardiac tamponade. This case highlights the importance of maintaining prolonged follow-up for individuals undergoing percutaneous ASD closure, given the possibility of potentially fatal late complications of ASD occlusion devices.

## Introduction

Atrial septal defect (ASD) is the second most common congenital heart defect (CHD) in adults, accounting for approximately 10% to 15% of all CHD cases [1]. There are four types of ASD, and while all of them can be surgically closed, only the ostium secundum (OS) defect can be percutaneously closed. Thus, in recent years, the use of percutaneously implanted occluding devices for the closure of ASD has become an increasingly popular alternative to surgical closure for patients with suitable anatomy, due to its minimally invasive nature and reduced rate of early complications and length of stay [2]. However, these devices are associated with potential complications, both early and late [3,4,5,6].

Early complications following ASD occlusion can occur during the procedure or in the immediate post-procedural period and include device malposition, embolization, air embolism, cardiac perforation, and arrhythmia [3,4,5,6,7].

Several late complications have been reported in the literature, including residual shunt, device erosion, device migration, late device fracture, thrombus formation, infective endocarditis, and atrial arrhythmias [3,4,5,6,8]. Device erosion can occur months to years after the device is implanted and can result in fistula formation, pericardial effusion, tamponade, and even death [9].

Overall, while the majority of patients who undergo ASD occluder placement do well, it is important to be aware of the potential late complications and closely monitor patients for any signs or symptoms of these complications.

## Case presentation

This case refers to a 48-year-old woman. At the age of 36 years, she was diagnosed with OS ASD, with a large left-to-right shunt (Qp:Qs = 1.9). Transcatheter ASD closure with an 18-mm Figulla Flex II ASD Occluder (Occlutech GmbH, Helsingborg, Sweden) was performed, resulting in complete shunt obliteration. The post-procedure period was uneventful. The patients had no other relevant past medical history.

Twelve years later, she resorted to the emergency department complaining of sudden onset chest pain, malaise, and nausea. On admission, she was hemodynamically stable, yet still complaining of chest pain. On auscultation, she was found to have muffled heart sounds, with clear lung fields bilaterally. Her initial blood tests were unremarkable. An electrocardiogram (ECG) showed sinus tachycardia, with no other relevant abnormalities. Arterial blood gas analysis showed metabolic acidosis with elevated lactates. Thoracoabdominopelvic computerized tomography (CT; Video 1) revealed a moderate-sized pericardial effusion and no evidence of pulmonary embolism or acute aortic dissection. The ASD closure seemed to be appropriately positioned.

**Video 1:** Thoracoabdominopelvic CT revealing a moderate pericardial effusion and no evidence of pulmonary embolism or acute aortic dissection. CT, computerized tomography

Immediately following this exam, the patient evolved unfavorably, developing signs of hemodynamic instability, with hypotension, tachycardia, and elevated jugular venous pressure. Point-of-care ultrasonography revealed a mild-to-moderate pericardial effusion with signs of hemodynamic compromise (Video 2). Emergency pericardiocentesis was performed, and less than 50 mL of hematic pericardial fluid was drained, resulting in significant hemodynamic improvement.

**Video 2:** Point-of-care ultrasonography, an apical four-chamber and subcostal view, showing a small pericardial effusion with signs of hemodynamic compromise.

**Figure 1:**
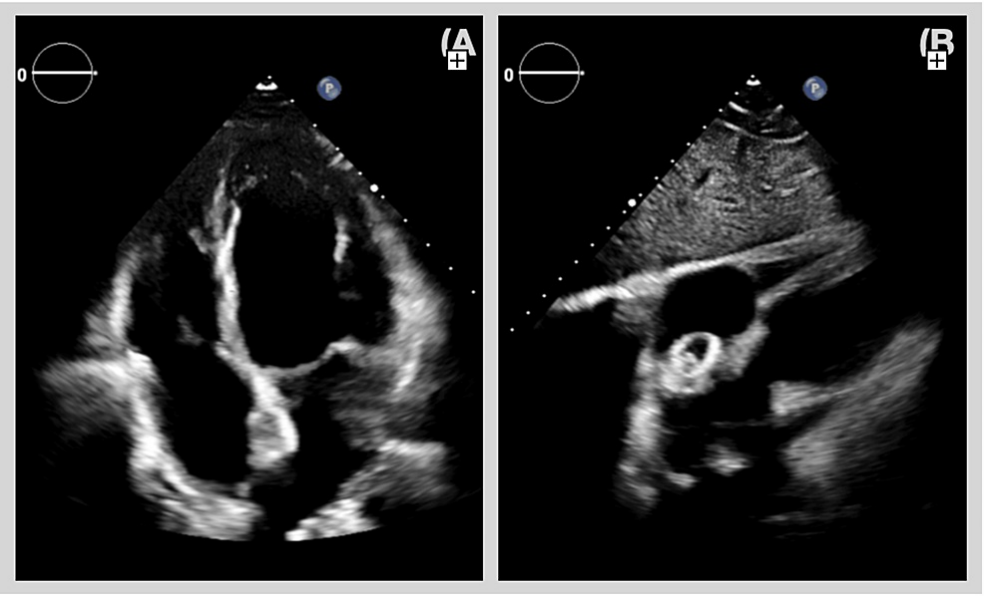
Transthoracic echocardiography: (A) apical four-chamber view and (B) subcostal view, showing an atrial septal occluder device.

After initial patient stabilization, a complete transthoracic echocardiogram (TTE; Figure 1) was performed, showing the occluder device in the interatrial septum, valvular apparatus without significant morphological changes, preserved biventricular systolic function, and small pericardial effusion, without collapse of cavities and normal respiratory variation of intracardiac flows.

Contrast thoracic CT showed a small-volume pericardial effusion (maximum thickness of 12 mm) and mild densification and edema of the surrounding fat, likely associated with the recent procedure, and with no evidence of contrast extravasation into the pericardium.

The patient remained under surveillance in the cardiac care unit for the next couple of days, and her clinical status improved progressively.

Transesophageal echocardiography (TEE; Video 3) showed that the edge of the ASD closure device was near the roof of the atria, with a nearby pericardial effusion (5-6 mm of larger dimensions). No passage of ultrasound contrast into the pericardial space was observed.

**Video 3:** Transesophageal echocardiography showing the edge of the ASD closure device in close proximity to the roof of the atria, with a nearby pericardial effusion (5-6 mm of larger dimensions). ASD, atrial septal defect

The exact cause for the hemopericardium was yet to be determined. Therefore, after a multidisciplinary discussion with cardiothoracic surgery, it was decided to proceed with exploratory surgery in an attempt to determine and eventually correct the cause of the cardiac tamponade.

**Figure 2:**
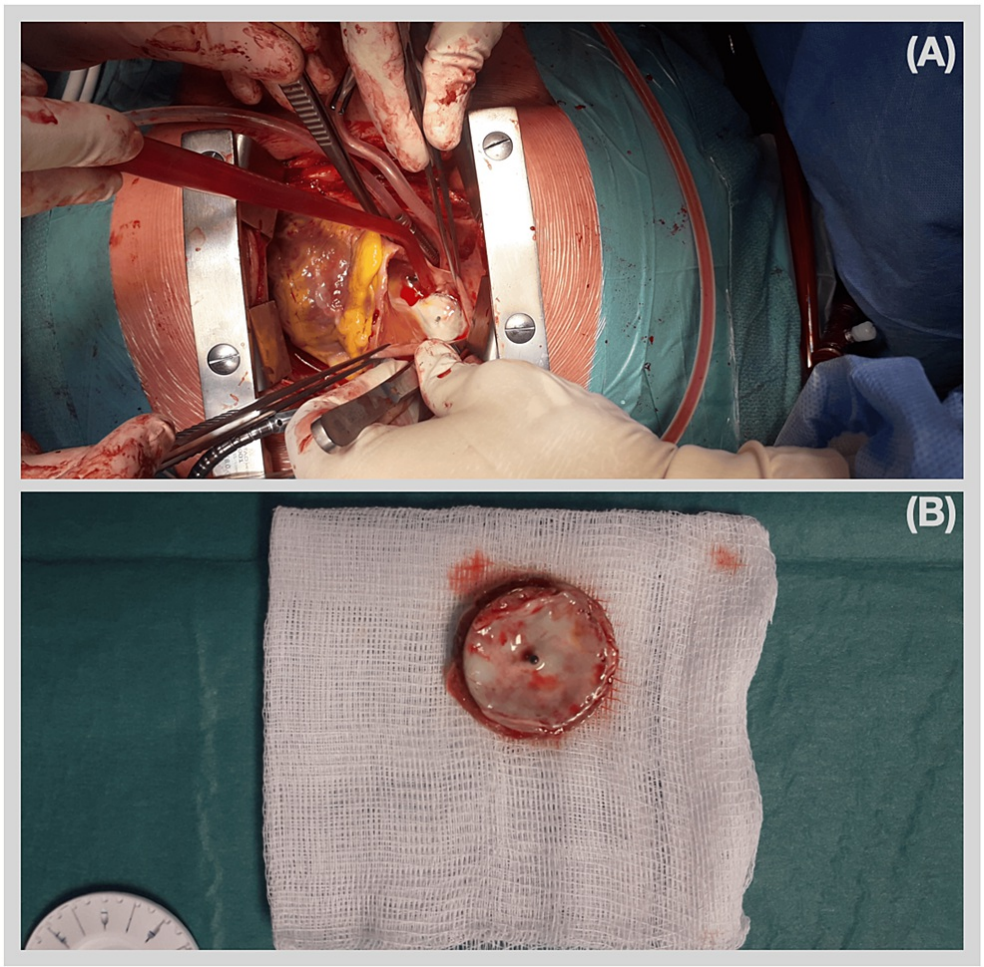
(A) Intraoperative photograph showing the device protruding through the right atrium wall with an already healed thickening; (B) explanted ASD occlusion device. ASD, atrial septal defect

Elective surgical exploration revealed a minor protrusion of the device through the right atrium wall, accompanied by a healed thickening. The device was then explanted, the right atrium wall was repaired, and the ASD was closed using a bovine pericardial patch (Figure 2). The postoperative period was uneventful, and the patient was discharged home five days after the surgery.

At the cardiology reevaluation visit, the patient was asymptomatic. A reevaluation echocardiogram revealed minimal pericardial effusion and no apparent residual shunts.

## Discussion

In recent years, the use of percutaneously implanted occluding devices for the closure of OS ASD has become an increasingly popular alternative to surgical closure for patients with suitable anatomy, due to its minimally invasive nature and reduced rate of early complications and length of stay [2,3]. However, these devices can be associated with late complications, including residual shunt, device erosion, device migration, late device fracture, thrombus formation, infective endocarditis, and atrial arrhythmias [3,4,5,6,8].

Device erosion has been reported as the second most common adverse effect of percutaneous closure of OS ASD with Amplatzer occluder devices, following embolization [10]. This complication is particularly concerning as it can be an urgent, life-threatening event. Although the majority were reported within the first six months, erosions continued to be reported as late as three years after deployment [10]. Diagnosing this complication poses a significant challenge, given its nonspecific clinical presentation, which spans from asymptomatic cases to those manifesting as cardiac tamponade with hemodynamic instability. Furthermore, the absence of specific diagnostic methods further complicates the diagnostic process. The most reliable diagnostic approach continues to be the diagnostic sternotomy, which should not be deferred in cases of elevated clinical suspicion and when other diagnostic methods prove inadequate in establishing a definitive diagnosis [11].

Reported risk factors for device erosion include an absent or deficient aortic rim, device protrusion into the atrial or aortic wall (or both), flaring of the device around the aortic root, device oversizing, and the occurrence of early pericardial effusion. However, there is still insufficient evidence to confidently determine which patient subgroups are at an increased risk for device erosion [11]. Thus, all patients undergoing percutaneous OS ASD closure require long-term follow-up, with regular clinical and echocardiographic assessments. Increased frequency is advisable in the first year postprocedure, due to the higher risk of complications during this period. Subsequently, long-term monitoring should continue with annual consultations and transthoracic echocardiography [10,12].

Therefore, while the majority of patients who undergo ASD occluder placement do well, it is important to be aware of the potential late complications and closely monitor patients for any signs or symptoms of these complications. Careful patient selection, device sizing, and placement techniques are crucial to minimize the risk of complications. A high index of suspicion is crucial for the timely identification and management of this potentially fatal complication.

## Conclusions

This case underscores the importance of vigilance and long-term follow-up in patients who undergo percutaneous ASD closure. In addition, it reiterates the need for a high level of suspicion to ensure a timely diagnosis of this type of complication. While the use of occluding devices has become a favored option due to its minimally invasive nature and reduced early complications, our experience with this patient highlights the necessity of ongoing surveillance for potential late complications.
